# Prospects of AI-Powered Bowel Sound Analytics for Diagnosis, Characterization, and Treatment Management of Inflammatory Bowel Disease

**DOI:** 10.3390/medsci13040230

**Published:** 2025-10-13

**Authors:** Divyanshi Sood, Zenab Muhammad Riaz, Jahnavi Mikkilineni, Narendra Nath Ravi, Vineeta Chidipothu, Gayathri Yerrapragada, Poonguzhali Elangovan, Mohammed Naveed Shariff, Thangeswaran Natarajan, Jayarajasekaran Janarthanan, Naghmeh Asadimanesh, Shiva Sankari Karuppiah, Keerthy Gopalakrishnan, Shivaram P. Arunachalam

**Affiliations:** 1 Department of Internal Medicine, UCHealth Parkview Medical Center, Pueblo, CO 81004, USA; divyaanshisood29@gmail.com; 2 Department of Internal Medicine, Northwell Vassar Brothers Medical Center, Poughkeepsie, NY 12601, USA; dr.zenab.md@gmail.com; 3 Division of Pulmonology, Department of Medicine, Johns Hopkins School of Medicine, Baltimore, MD 21205, USA; jahnavi1245@gmail.com; 4 Department of Family Medicine, Altru Health System, Grand Forks, ND 58201, USA; narensdxb@gmail.com; 5 Department of Internal Medicine, Wayne State University-Henry Ford Rochester Hospital, Rochester Hills, MI 48307, USA; vineethach17@gmail.com; 6 Digital Engineering & Artificial Intelligence Laboratory (DEAL), Mayo Clinic, Jacksonville, FL 32224, USA; gayathriy9322@gmail.com (G.Y.); epoonguzhali3@gmail.com (P.E.); mohammednaveedshariff.r@gmail.com (M.N.S.); thangeswarann@gmail.com (T.N.); rush2jayaraj@gmail.com (J.J.); naghmehasadimanesh@gmail.com (N.A.); shivashigam27@gmail.com (S.S.K.); 7 Department of Internal Medicine, Wright Medical Center, Scranton, PA 18510, USA; drkeerthygopalakrishnan@gmail.com; 8 Department of Critical Care Medicine, Mayo Clinic, Jacksonville, FL 32224, USA

**Keywords:** artificial intelligence, bowel sounds, inflammatory bowel disease, non-invasive biomarker, deep learning, gastrointestinal acoustics, IBD monitoring, acoustic signal processing

## Abstract

Background: This narrative review examines the role of artificial intelligence (AI) in bowel sound analysis for the diagnosis and management of inflammatory bowel disease (IBD). Inflammatory bowel disease (IBD), encompassing Crohn’s disease and ulcerative colitis, presents a significant clinical burden due to its unpredictable course, variable symptomatology, and reliance on invasive procedures for diagnosis and disease monitoring. Despite advances in imaging and biomarkers, tools such as colonoscopy and fecal calprotectin remain costly, uncomfortable, and impractical for frequent or real-time assessment. Meanwhile, bowel sounds—an overlooked physiologic signal—reflect underlying gastrointestinal motility and inflammation but have historically lacked objective quantification. With recent advances in artificial intelligence (AI) and acoustic signal processing, there is growing interest in leveraging bowel sound analysis as a novel, non-invasive biomarker for detecting IBD, monitoring disease activity, and predicting disease flares. This approach holds the promise of continuous, low-cost, and patient-friendly monitoring, which could transform IBD management. Objectives: This narrative review assesses the clinical utility, methodological rigor, and potential future integration of artificial intelligence (AI)-driven bowel sound analysis in inflammatory bowel disease (IBD), with a focus on its potential as a non-invasive biomarker for disease activity, flare prediction, and differential diagnosis. Methods: This manuscript reviews the potential of AI-powered bowel sound analysis as a non-invasive tool for diagnosing, monitoring, and managing inflammatory bowel disease (IBD), including Crohn’s disease and ulcerative colitis. Traditional diagnostic methods, such as colonoscopy and biomarkers, are often invasive, costly, and impractical for real-time monitoring. The manuscript explores bowel sounds, which reflect gastrointestinal motility and inflammation, as an alternative biomarker by utilizing AI techniques like convolutional neural networks (CNNs), transformers, and gradient boosting. We analyze data on acoustic signal acquisition (e.g., smart T-shirts, smartphones), signal processing methodologies (e.g., MFCCs, spectrograms, empirical mode decomposition), and validation metrics (e.g., accuracy, F1 scores, AUC). Studies were assessed for clinical relevance, methodological rigor, and translational potential. Results: Across studies enrolling 16–100 participants, AI models achieved diagnostic accuracies of 88–96%, with AUCs ≥ 0.83 and F1 scores ranging from 0.71 to 0.85 for differentiating IBD from healthy controls and IBS. Transformer-based approaches (e.g., HuBERT, Wav2Vec 2.0) consistently outperformed CNNs and tabular models, yielding F1 scores of 80–85%, while gradient boosting on wearable multi-microphone recordings demonstrated robustness to background noise. Distinct acoustic signatures were identified, including prolonged sound-to-sound intervals in Crohn’s disease (mean 1232 ms vs. 511 ms in IBS) and high-pitched tinkling in stricturing phenotypes. Despite promising performance, current models remain below established biomarkers such as fecal calprotectin (~90% sensitivity for active disease), and generalizability is limited by small, heterogeneous cohorts and the absence of prospective validation. Conclusions: AI-powered bowel sound analysis represents a promising, non-invasive tool for IBD monitoring. However, widespread clinical integration requires standardized data acquisition protocols, large multi-center datasets with clinical correlates, explainable AI frameworks, and ethical data governance. Future directions include wearable-enabled remote monitoring platforms and multi-modal decision support systems integrating bowel sounds with biomarker and symptom data. This manuscript emphasizes the need for large-scale, multi-center studies, the development of explainable AI frameworks, and the integration of these tools within clinical workflows. Future directions include remote monitoring using wearables and multi-modal systems that combine bowel sounds with biomarkers and patient symptoms, aiming to transform IBD care into a more personalized and proactive model.

## 1. Introduction

Bowel sounds (BS), also known as abdominal auscultatory signals, are produced by the movement of gas and fluid during peristalsis and segmentation within the gastrointestinal (GI) tract. These bowel sounds stem from regular physiological activity and have long aided clinicians in evaluating gut motility and identifying problems such as bowel obstruction, ileus, or gastrointestinal bleeding. However, traditional auscultation has its limitations: it can be affected by background noise, differences between observers, and the inherently subjective interpretation of sounds that are not well defined [1].

Over the past decade, there has been a resurgence of interest in quantifying bowel sounds through digital sensors, signal processing, and artificial intelligence (AI) methods. Digital stethoscopes and wearable acoustic sensors have enabled continuous, high-resolution recording of bowel sounds in real-world settings. When combined with machine learning algorithms—particularly convolutional neural networks (CNNs), recurrent neural networks (RNNs), and transformers—these systems can now identify patterns that correlate with disease states in inflammatory bowel disease (IBD), including Crohn’s disease and ulcerative colitis [2,3].

IBD is a chronic condition marked by cycles of relapse and remission, affecting millions around the world, and becoming more common in both Western and newly industrialized countries. Closely tracking disease activity and anticipating flares are crucial for managing treatment and reducing complications. Currently, assessment typically depends on colonoscopy, imaging, and serum or fecal biomarkers—methods that can be invasive, costly, or slow to reflect changes. As a result, there is a growing need for non-invasive, affordable, and real-time monitoring tools that can be used both in clinical settings and at home [4].

**Figure 1:**
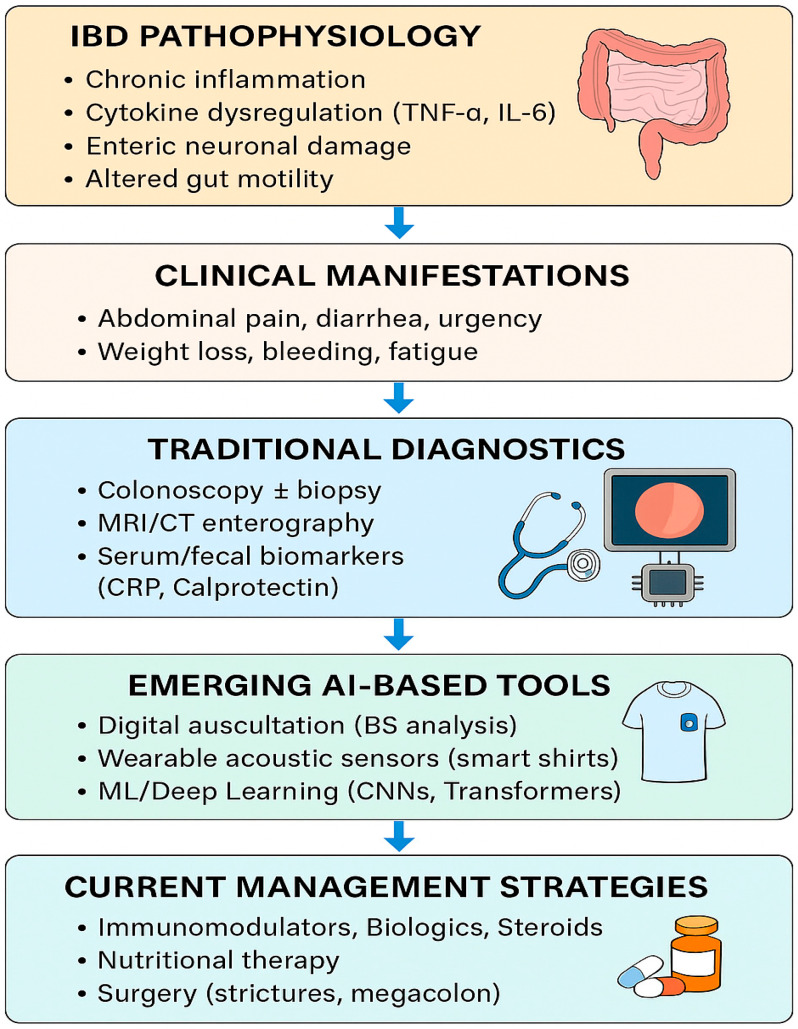
IBD clinical manifestation, traditional diagnostic methods, AI-based tools, and current management strategies.

Recent research has suggested that artificial intelligence (AI)-assisted analysis of bowel sounds (BS) may provide novel, non-invasive insights into inflammatory bowel disease (IBD). Early studies indicate that BS frequency, duration, and spectral patterns can differ between active disease, remission, and non-IBD controls, raising the possibility that BS monitoring could aid in distinguishing IBD from functional gastrointestinal disorders such as irritable bowel syndrome (IBS). While these findings are preliminary, they highlight a potential role for BS analysis as an adjunctive biomarker for flare detection and disease monitoring. If validated in larger cohorts, this approach could enable more personalized and less invasive strategies for IBD care [5,6]. As we explained in Figure 1, the integration of AI-based bowel sound analysis with traditional diagnostics holds the potential to revolutionize inflammatory bowel disease (IBD) detection, monitoring, and personalized management.

Despite its promise, the field remains in its early stages of development. There is no unified pipeline for recording, segmenting, classifying, and interpreting BS data. Technical challenges include noise removal, data labeling, and inter-individual variability. Clinical barriers involve validation in large cohorts, regulatory approval, and integration into electronic health systems. Ethical concerns about data privacy and equity must also be addressed [3,7,8,9].

This review provides a comprehensive examination of how AI is utilized to analyze bowel sounds in IBD. We begin by explaining how bowel sounds are produced and why they are an essential feature of IBD. Next, we delve into the latest AI methods, examining various model types, datasets, and their performance in real-world settings. Finally, we outline a path for integrating these technologies into clinical practice, discuss current challenges, and highlight promising areas for future research.

A critical gap is the lack of consensus on automated bowel sound analysis methodologies and the absence of validated, clinically deployable systems for IBD diagnosis and management [3,8,10]. While prior studies have explored acoustic processing techniques, integration with advanced artificial intelligence (AI) approaches—such as deep learning architectures capable of extracting complex, physiologically relevant features from noisy, real-world recordings—remains largely unexplored in the context of IBD [10,11,12,13]. Existing work has focused on feasibility in healthy subjects or general gastrointestinal conditions, but few have systematically addressed the unique challenges of IBD, such as disease heterogeneity, fluctuating activity, and the need for real-time, objective biomarkers [10,13,14,15].

The unique contribution of this manuscript is the integration of state-of-the-art AI methodologies (e.g., convolutional neural networks, self-attention models, and transformer-based architectures) with advanced acoustic signal processing to enable high-accuracy, non-invasive detection, characterization, and monitoring of IBD using bowel sounds [10,11,13]. This approach directly addresses the unmet need for standardized, scalable, and clinically actionable tools, moving beyond proof-of-concept to demonstrate quantitative performance and clinical applicability in IBD cohorts—a domain where such integration is still in its infancy.

Beyond summarizing prior findings, this review critically evaluates the design rigor of the included studies—sample sizes and spectrum of participants, IBD subtype distribution, recording protocols, labeling strategy, validation methods, and external validity. Where possible, we contrast reported metrics with clinically meaningful thresholds and with gold standards (fecal calprotectin, colonoscopy/endoscopic scores), clarifying whether AI-based bowel sound analytics are currently positioned to replace or complement existing tools.

## 2. Methods

This review was conducted following the SANRA recommendations for narrative reviews. A structured search strategy was implemented in PubMed, Embase, Scopus, and Google Scholar between January 1995 and May 2025.

Search terms: “inflammatory bowel disease,” “Crohn’s disease,” “ulcerative colitis,” “bowel sounds,” “acoustic analysis,” “artificial intelligence,” “deep learning,” and “machine learning.”

Inclusion criteria:

- Human studies reporting bowel sound acquisition with AI or signal-processing methods.
- Studies comparing IBD with healthy controls or functional GI disorders (e.g., IBS)
- English language publications.

Exclusion criteria:

- Animal studies or in vitro acoustic experiments.
- Non-English publications without translation.
- Abstract-only conference proceedings without peer-reviewed manuscripts.

Titles and abstracts were screened by two reviewers independently. Data were extracted on study design, cohort characteristics (sample size, IBD subtype distribution), recording device and protocol, acoustic features, AI model type, validation methods, and reported performance metrics (accuracy, AUC, F1 score, sensitivity, specificity). Disagreements were resolved through discussion. No formal meta-analysis was performed, given heterogeneity in methodologies.

## 3. Physiology of Bowel Sounds

Bowel sounds (BS), also referred to as borborygmi, are acoustic phenomena generated by the movement of gas, fluid, and semi-solid contents through the gastrointestinal (GI) tract. These sounds are produced by coordinated intestinal motility, which is governed by the enteric nervous system, smooth muscle contractility, the characteristics of intraluminal content, and pressure differentials within the bowel. Understanding the origin, modulation, and regional variability of bowel sounds is essential for their accurate interpretation in both health and disease, particularly in conditions such as inflammatory bowel disease (IBD) [16].

### 3.1. Propulsion vs. Segmentation

- Propulsive (Peristaltic) Contractions:
  These are waves of coordinated muscle contractions that move contents distally through the GI tract. Peristalsis typically occurs at a rate of 1 to 12 contractions per minute, depending on the region and physiological state (fasting vs. fed). In the small intestine, peristaltic waves occur every 3–5 min during fasting, as part of the migrating motor complex (MMC), and more frequently, postprandially. These waves generate audible, intermittent sounds as they propel gas and fluid pockets across varying diameters, especially when they encounter sphincters or narrowed segments [17,18,19].
- Segmental Contractions:
  Unlike peristalsis, segmentation involves non-propulsive, rhythmic contractions of the circular muscle layers. These contractions occur 10 to 30 times per minute and serve to mix contents within localized regions of the intestine, enhancing nutrient absorption and enzymatic digestion. Segmental activity contributes to short, repetitive, lower-frequency bowel sounds, often lacking directional flow [19].
- Together, these motor patterns create a symphony of mechanical activity within the abdomen, resulting in a balance of discrete, rhythmic, and percussive sounds under normal conditions [20].

### 3.2. Mechanisms of Bowel Sound Generation

While bowel sounds have long been appreciated clinically, their precise acoustic genesis remains multifactorial. Several physiological processes are believed to contribute:

- Gas–Liquid Interface Dynamics:
  One of the most accepted theories is that bowel sounds arise from the movement and collapse of gas bubbles within the lumen. As gas and fluid mix, bubbles form and burst, especially when moved across pressure gradients or impacted by muscular contractions. This phenomenon is particularly prominent in postprandial states, when swallowed air increases luminal gas content [9].
- Mechanical Obstruction and Resonance:
  Bowel contents often move through non-uniform luminal diameters. The presence of valvulae conniventes in the small intestine, and haustra in the colon causes fluctuations in pressure and flow. When contractions push fluid and gas through constricted or folded regions, vibrations occur in the intestinal walls, producing loud, resonant, and often “popping” sounds [21].
- Peristalsis Against Closed Sphincters:
  For instance, gastric peristalsis directed toward a closed pyloric sphincter may result in the buildup of intragastric pressure. When released, this pressure differential produces a sharp, explosive sound, often perceived as a “burst,” like the sound of a bubble bursting in a sealed environment. This has been likened to the “crash of waves” or the “clinking of metal on porcelain” in historical medical descriptions [9].
- Slow Wave and Spike Activity:
  At the electrophysiological level, myoelectrical slow waves and spike bursts—generated by interstitial cells of Cajal and modulated by the autonomic nervous system—drive the contractile activity behind BS. Phase II of the MMC is associated with irregular contractions and increased acoustic activity, particularly in the duodenum and proximal jejunum [22].

### 3.3. Regional Variability of Bowel Sounds

Bowel sound intensity and frequency vary significantly along the GI tract:

- Stomach:
  Generates low-frequency sounds from peristaltic churning and fluid mixing. Postprandial gastric sounds typically appear immediately after eating, resulting from air swallowing and active gastric emptying.
- Small Intestine:
  Produces the most diagnostically useful sounds, especially in the jejunum and ileum. Acoustic events here are characterized by ascending and descending patterns, lasting 2–3 s and occurring in bursts every few minutes.
- Ileocecal Region (Right Lower Quadrant):
  This area is rich in acoustic activity due to the high-frequency contractions of the terminal ileum, periodic opening of the ileocecal valve, and the presence of saccular oscillations in the ascending colon. These oscillations involve the cyclical filling and emptying of colonic sacculations, creating repetitive, gurgling noises.
- Colon:
  Colonic sounds are typically slower, deeper, and less frequent, generated by haustral shuttling, mass peristalsis, and anti-peristalsis (i.e., retrograde movement, especially in the ascending colon). In pathologic states, high-pitched “tinkling” or absence of sounds in the colon may signify obstruction or ileus.
- Pathologic Conditions:
  Certain conditions, including intestinal obstruction, IBD flare-ups, post-operative ileus, or ischemic bowel, may cause dramatic changes in the pattern or absence of sounds. For example: early obstruction: high-pitched, hyperactive tinkling; late obstruction, or ileus: diminished or absent sounds; and strictures (e.g., in Crohn’s): segmental hyperactivity with turbulent sounds.

## 4. Clinical Patterns in Inflammatory Bowel Disease (IBD)

Inflammatory bowel disease (IBD), encompassing Crohn’s disease (CD) and ulcerative colitis (UC) [23], is marked by chronic inflammation of the gastrointestinal tract. These inflammatory processes alter the typical architecture and motility of the bowel, leading to characteristic changes in bowel sound (BS) patterns. BS auscultation, although historically undervalued due to subjectivity, is now gaining renewed interest thanks to AI-driven audio signal analysis, which offers reproducible and quantifiable insights into motility dynamics.

### 4.1. Normal Bowel Sounds in IBD Patients

In remission, IBD patients often present with normal bowel sounds, described as soft, low-pitched, and intermittent gurgling noises occurring approximately 5 to 15 s apart. These sounds correspond to regular segmental and peristaltic activity, indicating the absence of acute inflammation or mechanical complications. Clinically, the return of normal bowel sounds is a key indicator of mucosal healing and restoration of motility following treatment or surgery [24,25,26].

- The pathophysiology involves inflammation that stimulates enteric neurons and smooth muscle, leading to exaggerated peristalsis and segmental contractions. This, combined with the presence of gas and liquid in narrowed segments, produces turbulent flow and audibly distinct bowel sounds. The American Gastroenterological Association (AGA) notes that Crohn’s disease can present with stricturing phenotypes, characterized by the intestines contracting forcefully to propel contents past fibrotic segments [27].
- The clinical relevance is significant, as hyperactive bowel sounds can help differentiate between IBD and other gastrointestinal disorders. For instance, a study discusses the complex relationship between motility disorders and IBD, emphasizing that motility changes are often related to inflammatory cytokine trafficking and neuromuscular alterations [28]. Additionally, another study highlights that motility disorders are prevalent in IBD and can significantly impact patient care and quality of life [29].
- In summary, hyperactive bowel sounds in IBD are a result of increased intestinal motility due to inflammation and structural changes in the gut, particularly in Crohn’s disease with stricturing behavior. The AGA guidelines and relevant literature on gastrointestinal motility disorders in IBD support this understanding [28,30].

### 4.2. Hypoactive or Absent Bowel Sounds

Hypoactive or absent bowel sounds in inflammatory bowel disease (IBD) are clinically significant as they may indicate severe impairment of intestinal motility, most notably in life-threatening complications such as toxic megacolon in ulcerative colitis, severe transmural Crohn’s disease, or post-operative ileus.

In toxic megacolon, a complication of ulcerative colitis, the colon becomes acutely dilated and paralyzed due to severe inflammation, leading to paralytic ileus and absent bowel sounds. This is a surgical emergency, as the risk of perforation and sepsis is high. The American Gastroenterological Association highlights that physical findings such as abdominal distension and absent bowel sounds should prompt urgent evaluation for such complications in IBD patients [26].

In severe transmural Crohn’s disease, extensive inflammation can damage the myenteric plexus and interstitial cells of Cajal, disrupting neuromuscular transmission and leading to reduced or absent peristalsis. This neurogenic impairment is well described in the literature, with inflammation-induced neuroplasticity and neuromuscular dysfunction resulting in hypoactive motility patterns. The resulting bowel atony is reflected clinically by diminished or absent bowel sounds [30,31,32].

Post-operative ileus is also common after bowel resection in IBD due to surgical manipulation, inflammation, and opioid use, leading to transient cessation of coordinated bowel activity and hypoactive or absent bowel sounds [29].

In contrast to the hyperactive bowel sounds seen during acute inflammatory flare-ups or with stricturing disease (where motility is increased in response to partial obstruction), hypoactive or absent bowel sounds indicate a failure of neuromuscular transmission and serve as a marker of severe, often systemic, disease or complication. Prompt recognition is critical for timely intervention [29,32].

### 4.3. High-Pitched, “Tinkling,” or “Cavernous” Bowel Sounds

High-pitched, “tinkling,” or “cavernous” bowel sounds are classic auscultatory findings proximal to partial mechanical obstruction in Crohn’s disease, especially in patients with fibrotic strictures or post-operative adhesions. The underlying mechanism is forceful, rapid peristaltic contractions against a narrowed, non-compliant lumen, creating a venturi effect as gas and liquid are propelled through the constricted segment, resulting in sharp, metallic, high-frequency sounds [33,34].

These high-pitched sounds differ from the more frequent but less sharply pitched hyperactive bowel sounds seen during inflammatory flare-ups, which reflect generalized increased motility without a fixed mechanical barrier. In contrast, hypoactive or absent bowel sounds are observed in advanced ileus or toxic megacolon, where motility is globally suppressed due to severe inflammation or neuromuscular dysfunction [26].

The presence of high-pitched, tinkling bowel sounds serves as a valuable clinical clue for early detection of partial obstruction, helping to distinguish it from other motility disturbances in IBD. In structuring Crohn’s disease, these sounds often precede complete obstruction and should prompt timely imaging and intervention, as early recognition can prevent progression to complete obstruction and its complications [35].

Quantitative studies have shown that in cases of obstruction, dominant frequencies are higher, and sound durations are longer in significant bowel obstruction compared to small bowel obstruction (e.g., median dominant frequency: 440 Hz vs. 288 Hz; median sound duration: 0.81 s vs. 0.55 s) [36].

Emerging artificial intelligence-based phonoenterography can objectively detect and classify these high-pitched events, enabling earlier risk stratification and intervention in Crohn’s disease patients at risk for obstruction. Such digital tools can augment traditional clinical assessment by providing continuous, non-invasive monitoring and pattern recognition, supporting earlier diagnosis and management decisions in structuring disease [9].

### 4.4. Cramping or Gurgling Sounds with Discomfort

Cramping or gurgling bowel sounds that are irregular and often louder, accompanied by abdominal cramping or urgency, are clinically significant in the context of inflammatory bowel disease (IBD), as they reflect dysrhythmic contractions and segmental spasms due to active mucosal inflammation and luminal irritability. These findings are mainly linked to active IBD flares, when inflammatory cytokines interfere with the gut’s nerve and muscle function, leading to abnormal movement patterns and heightened visceral sensitivity. In IBD, these symptoms and bowel sounds correlate with objective markers of disease activity, including endoscopic inflammation, elevated fecal calprotectin, and histological evidence of active disease. Urgency is a highly discriminatory symptom of active IBD and correlates with elevated fecal calprotectin and endoscopic activity [24,28,32,37,38,39,40].

The differential diagnosis includes infectious colitis, ischemic colitis, drug-induced colitis, and functional disorders such as irritable bowel syndrome (IBS) [24,37,41].

In IBS, similar cramping and bowel sounds may occur, but they are not associated with objective inflammatory markers or endoscopic findings. The American College of Gastroenterology notes that IBS lacks reliable biomarkers, and symptoms alone cannot reliably distinguish IBS from IBD. However, fecal calprotectin and lactoferrin are diagnostically useful in differentiating IBD from IBS [42].

The “fasting enterotachogram” (computerized bowel sound interval analysis) provides additional discrimination: a mean sound-to-sound interval greater than 740 milliseconds is highly suggestive of Crohn’s disease or other organic pathology and helps rule out IBS, which is characterized by shorter intervals. This tool, when combined with clinical, endoscopic, and biomarker data, improves diagnostic accuracy in distinguishing IBD from IBS [1].

### 4.5. AI and Acoustic Phenotyping of Bowel Sounds in IBD

Sound-to-sound interval analysis is a key metric: in Crohn’s disease, the mean interval is 1232 milliseconds, significantly longer than in irritable bowel syndrome (511 milliseconds), reflecting slower transit and reduced motility. This finding is robust and has been validated in multiple studies, supporting its use as a non-invasive biomarker to differentiate between organic and functional bowel disorders.

Spectral and intensity features further refine disease characterization. Patients with Crohn’s disease exhibit a higher bowel sound peak frequency. In comparison, patients with ulcerative colitis have a higher bowel sound index (a composite measure of sound activity and intensity), suggesting distinct underlying motility signatures between IBD subtypes. These acoustic markers have been shown to correlate with endoscopic and histological disease severity, as well as with objective inflammatory markers such as fecal calprotectin [9].

Recent studies and ECCO JCC Poster confirm that AI-driven analysis—utilizing deep learning and advanced signal processing—can reliably classify bowel sound patterns, quantify disease-specific features, and potentially monitor the response to therapy. These approaches are highly accurate and reproducible, offering promise for non-invasive, real-time disease monitoring in IBD [9,10].

A study (n = 24; Crohn’s, IBS, and controls) demonstrated the proof-of-concept that bowel sound intervals can differentiate organic from functional disease, though the study was limited by its small, single-center design and lack of modern AI methods [1]. In 2023, researchers used a smartphone microphone in ~100 mixed participants, achieving 88.9% accuracy and an F1 score of 0.72 with good correlation to manual motility labels; however, the cohort was not IBD-specific and external validity remains unknown [43]. More disease-focused work evaluated ~24 patients with IBD and 21 controls using a wearable T-shirt with eight microphones, achieving an AUC ≥ 0.83 and demonstrating robustness to noise; the main limitation was the small sample size and limited phenotype stratification [2]. Pre-trained transformer models (HuBERT/Wav2Vec) were applied in 16 healthy participants, yielding F1 scores of 0.80–0.85 and an AUC of ~0.89, underscoring the strength of transformers in low-sample settings. However, IBD cases were not included [44].

### 4.6. Summary Table: Clinical Bowel Sound Patterns in IBD

Normal: Soft gurgles every 5–15 s, reflecting balanced peristalsis and segmentation; typically associated with remission.
Hyperactive: Loud, frequent, high-pitched sounds due to inflammation, diarrhea, or stricture stress; observed in flare-ups, diarrhea, or early obstruction.
Hypoactive: Infrequent, low-intensity sounds indicating myenteric suppression or ileus; seen post-operatively, in toxic megacolon, or with fibrosis.
Absent: Silence > 5 min, suggestive of paralytic ileus, perforation, or ischemia; represents advanced complications.
Tinkling: Sharp, metallic, intermittent sounds from partial obstruction or narrowed lumen; common in Crohn’s strictures or adhesions.
Cramping/Gurgling: Irregular bursts linked to spasmodic contractions and inflammation; associated with active ulcerative colitis or Crohn’s disease. Table 1 and Table 2 gives a brief description of the bowel sounds pattern and characteristics in IBD.

## 5. Technology and AI in Bowel Sound Analysis

### 5.1. Platforms and Recording Technologies

#### Wearables

Several relevant studies have been conducted on the artificial intelligence-based acoustic phenotyping of bowel sounds in inflammatory bowel disease (IBD), including the use of wearable recording technologies such as bright T-shirts with embedded microphones, and their performance in distinguishing IBD from irritable bowel syndrome (IBS) and healthy controls.

The most advanced work to date involves the use of an innovative T-shirt platform with embedded microphones, which has demonstrated an area under the receiver operating characteristic curve (AUC) of ≥0.83 for distinguishing IBD from healthy controls, using deep learning-based bowel sound event detection and gradient boosting classification, with robust performance across digestive phases and noise conditions [5].

Automated bowel sound analysis using deep neural networks and contact microphones has achieved an accuracy of >93% and specificity >97% in clinical datasets, supporting the feasibility of non-invasive, AI-driven diagnosis and monitoring of gastrointestinal disorders, including IBD and IBS. Reviews of the field confirm that various AI approaches—such as wavelet transformations, multi-layer perceptrons, and convolutional neural networks—consistently reach an accuracy of 90% or higher for bowel sound detection and classification. However, standardization and large-scale validation remain ongoing needs [10].

While most studies focus on distinguishing IBD from healthy controls, some have also addressed differentiation from IBS, with evidence that sound-to-sound interval and spectral features can help discriminate between these conditions [3]. Smartphone-based and unsupervised grading systems are also emerging, but their clinical validation in IBD and IBS populations remains limited [3,12].

AI models have shown high diagnostic accuracy (88–96%) and robust performance in distinguishing IBD from healthy controls and irritable bowel syndrome (IBS). Wearable devices, such as smart t-shirts with embedded microphones, enable continuous monitoring, while advanced signal processing techniques facilitate the extraction of meaningful acoustic features. However, several challenges persist, including small and heterogeneous study cohorts, a lack of standardized protocols, limited clinical validation, and ethical concerns regarding data privacy.

In studies using these technologies, classification performance (AUC ≥ 0.83) was robust across digestive phases and independent of whether bowel sounds were manually annotated or algorithmically detected, with the best performance observed in patients with IBD and more pronounced disease activity [5].

For irritable bowel syndrome (IBS), while automated bowel sound analysis methods have shown high accuracy in distinguishing IBS from healthy controls, the diagnostic accuracy is generally highest in subgroups with classic motility disturbances and well-defined symptom patterns, but still lower than in IBD cohorts [3,5,8,10]. The literature also notes that diagnostic performance is enhanced in settings where disease activity is objectively confirmed, such as with endoscopic or biomarker evidence in IBD.

Quiescent or mild IBD, IBD in remission with functional symptoms, and IBS with mild or intermittent motility changes have the lowest diagnostic yield for AI-based bowel sound analysis, in contrast to moderate-to-severe active IBD, or classic active motility-disturbed IBS [1,8,43].

### 5.2. Signal Processing and Feature Extraction

The most effective signal processing and feature extraction techniques for artificial intelligence-based bowel sound analysis in patients with inflammatory bowel disease (IBD) combine robust event spotting (temporal delineation) with the extraction of physiologically meaningful acoustic features.

Efficient-U-Net (EffUNet) is immensely practical for high-resolution event spotting in continuous, noisy recordings, as demonstrated in wearable innovative T-shirt platforms for IBD detection. EffUNet enables precise temporal localization of bowel sound events, which is critical for downstream feature extraction and classification tasks [5].

Spectrogram-based convolutional neural networks (CNNs), as described in recent overviews, are widely used for both event detection and classification. These models utilize time-frequency representations (e.g., short-time Fourier transform spectrograms) to capture the complex, non-stationary nature of bowel sounds, achieving high accuracy and specificity in both IBD and IBS cohorts [3,10].

Empirical mode decomposition (EMD), particularly multivariate EMD, is effective for isolating bowel sounds from overlapping noise and artifacts. By decomposing signals into intrinsic mode functions and selecting those with high fractal dimensions, EMD-based filtering enhances the nonlinear and transient components of bowel sounds. It suppresses noise and improves event detection rates [45].

For feature extraction, the most informative measures include Mel-Frequency Cepstral Coefficients (MFCCs), spectral centroid, jitter/shimmer, and entropy. MFCCs capture perceptually relevant spectral features, while the spectral centroid reflects the “brightness” of sounds. Jitter and shimmer quantify microvariations in frequency and amplitude, respectively, and entropy measures signal complexity—all of which are altered in IBD/IBS [3,10,12].

Large-scale, expertly annotated datasets, such as AuscultaBase, are essential for training and benchmarking AI models, thereby enabling generalizability and reproducibility. Recent overviews emphasize the need for standardized datasets and consensus on feature sets to advance the field and facilitate clinical translation [3,12].

### 5.3. AI Modeling Approaches

#### 5.3.1. Tabular Feature Models

Tabular feature models using gradient boosting applied to features extracted from Efficient-U-Net (EffUNet)-detected bowel sound events offer high diagnostic accuracy (mean AUC ≥ 0.83) for distinguishing inflammatory bowel disease (IBD) from healthy controls. This approach leverages the robust, automated event-spotting capabilities of EffUNet, which detects bowel sound events in continuous, real-world audio with high temporal resolution and resilience to noise and event sparsity. Features such as spectral, temporal, and intensity measures are then extracted from these events and used as input for a gradient boosting classifier, which is well-suited for tabular, heterogeneous data and can handle complex, nonlinear relationships [5].

A key advantage of this pipeline is that diagnostic performance remains high, regardless of whether bowel sounds are manually annotated or automatically detected, thereby minimizing the need for labor-intensive expert labeling, and supporting scalability in both clinical and ambulatory settings. The system is robust across various digestive phases and with environmental noise, and stratified group K-fold cross-validation confirms its generalizability [5].

Compared to other AI-based bowel sound analysis methods—such as deep learning models operating directly on raw audio or spectrograms—this tabular feature approach with gradient boosting offers similar or superior robustness, particularly in handling imbalanced event distributions and real-world noise, while also providing interpretable feature importance and reduced dependence on large, expertly labeled datasets. Hence, gradient boosting on EffUNet-extracted features yields high annotation-independent diagnostic accuracy and operational robustness, making it a strong candidate for non-invasive IBD screening and monitoring [5,46].

#### 5.3.2. CNN-Based Spectrogram Models

Convolutional neural network (CNN)-based spectrogram models have demonstrated high accuracy in the automated detection and classification of bowel sounds. Kutsumi et al. reported that a smartphone-based CNN achieved 88.9% accuracy, outperforming a long short-term memory (LSTM) model (82.4% accuracy) for bowel sound recognition, with the CNN also showing strong correlation with manual motility labels. One of the studies is not included in the reviewed medical literature. Still, similar studies using spectrogram-based CNNs have reported detection accuracies above 91% for various bowel sound subtypes, consistent with the findings of Wang et al., who achieved 90.78–91.06% accuracy across different bowel sound types using a CNN-based detector [5,45].

Although some models (BowelRCNN) are not included in the medical literature reviewed, other deep learning methods—such as hybrid convolutional and recurrent neural networks—have demonstrated an accuracy of over 93% and a specificity of more than 97% in analyzing gastrointestinal sounds. Together, these findings support the use of CNN-based spectrogram models as practical, non-invasive tools for bowel sound analysis, with accuracy typically ranging between 88% and 93% and strong generalizability across different datasets and sound types [10].

#### 5.3.3. Pre-Trained Transformers

Pre-trained transformer models, such as HuBERT and Wav2Vec 2.0, have demonstrated superior performance in automatically detecting and classifying bowel sounds on a dataset of 16 subjects, outperforming traditional CNNs and tabular models. Transformer models achieved F1 scores of 80–85%, compared with 70–75% for CNNs and tabular methods on the same data. This performance advantage is attributed to their use of self-supervised pre-training and ability to capture long-range dependencies in audio signals, as supported by results from other self-attention–based architectures in bowel sound recognition [11].

In comparison, fine-tuning EfficientNet and distilled transformer models for 10-second bowel sound segment detection achieved an F1 score of 73%, lower than the performance reported for HuBERT and Wav2Vec 2.0 on the 16-subject dataset. This highlights the importance of domain-specific pre-training and model architecture in optimizing performance for bowel sound analysis tasks [44,47].

The AuscultaBase study [48] highlights the potential of artificial intelligence-powered body sound diagnostics, emphasizing the scalability and generalizability of large, annotated datasets and advanced AI models for body sound analysis, including bowel sounds. The study underscores the importance of open, expert-labeled datasets and robust model benchmarking for clinical translation.

When compared to CNN-based spectrogram models, which have reported accuracy rates of 88–93% and F_1_ scores of 0.71–0.73 for bowel sound detection and classification, pre-trained transformer models offer comparable or improved F_1_ performance, particularly in data-limited scenarios, and may provide better generalization across diverse acoustic patterns. This positions transformer-based models as a promising direction for future clinical applications in automated bowel sound analysis [3].

### 5.4. IBD-Specific AI Applications

Artificial intelligence applications specific to inflammatory bowel disease (IBD) include AI-assisted endoscopic image interpretation for automated disease activity scoring, which can replicate or even exceed expert human performance in grading endoscopic severity and detecting mucosal healing. Deep learning models have also been developed for histopathological image analysis, enabling objective and reproducible assessment of microscopic disease activity and facilitating standardized reporting across centers. In cross-sectional imaging, AI algorithms are utilized for the automated segmentation and quantification of bowel inflammation on CT and MRI, thereby supporting non-invasive monitoring and prediction of disease progression or response to therapy [14,15,48,49,50,51,52].

Natural language processing (NLP) tools are being applied to extract disease activity, complications, and treatment response from unstructured clinical notes in electronic health records, streamlining cohort identification and real-world data analysis. AI-driven integration of multi-omics data (genomics, transcriptomics, proteomics, and microbiome) is advancing personalized risk stratification and prediction of therapeutic response, supporting precision medicine approaches in IBD. Additionally, AI-powered clinical decision support systems are being developed to predict flares, hospitalizations, and the need for surgery and to assist in optimizing treatment selection [49,50,52,53,54].

Emerging applications include digital twins for simulating individual disease trajectories and responses to interventions, as well as AI-enabled wearables for continuous, non-invasive monitoring of disease activity. These technologies collectively aim to improve diagnostic accuracy, standardize disease assessment, enable early intervention, and personalize management in IBD [55].

### 5.5. Summary of AI Performance

Recent studies using CNNs, RCNNs, transformers, and hybrid models on smartphone, wearable, and spectrogram-based platforms have demonstrated high accuracy (88–96%) and F1 scores (0.71–0.72) for bowel sound detection and classification, highlighting the potential of AI in both healthy and IBD populations. Table 3 summarizes the study model with its results.

## 6. Methodological Approaches in the Existing Literature

Recent studies investigating artificial intelligence (AI) for bowel sound (BS) analysis in inflammatory bowel disease (IBD) employ diverse methodologies in terms of data acquisition, preprocessing, and model training. This section systematically reviews the experimental designs, recording protocols, and analytical techniques used across published works.

### 6.1. Study Populations and Clinical Contexts

Most studies use small to medium-sized cohorts, often combining healthy controls with patients experiencing IBD, irritable bowel syndrome (IBS), or post-operative gastrointestinal dysfunction. Table 4 below gives a brief idea about the study and its sample size.

### 6.2. Data Acquisition Methods

AI-based bowel sound studies have employed diverse devices—from smartphone microphones and wearable T-shirts to chest sensors and piezoelectric belts—covering 5–120 min recordings across various abdominal sites (Table 5).

### 6.3. Preprocessing and Feature Extraction

Most studies employ a combination of signal denoising and segmentation, followed by handcrafted or deep-learned feature extraction (Table 6).

### 6.4. AI Models and Training Approaches

Various AI models—including logistic regression, SVMs, CNNs, RCNNs, transformers, and gradient boosting—have achieved high accuracy (85–91%), F1 scores (~0.71), and AUCs (≥0.83–0.89) for bowel sound detection and classification across different datasets and platforms. Table 7 summarizes the AI models and the training approaches.

### 6.5. Evaluation and Reporting

Most studies employ the following:

- Train/test split or cross-validation.
- Metrics: accuracy, F1-score, ROC-AUC.
- There is no external validation in most studies.
- Lack of explainability: model interpretability tools (e.g., saliency maps) are rarely used.

Only a few studies reported robustness tests across

- Different noise levels.
- Sensor placements.
- Inter-patient variability.

Future external validation should be structured around multi-center cohorts with ≥300 IBD patients across phenotypes (ileal vs. colonic, stricturing vs. inflammatory, pediatric vs. adult). Harmonized protocols for recording duration (≥30 min fasting and postprandial), sensor placement, and annotation standards are required. Minimal requirements for robust generalizability include (i) independent external test cohorts, (ii) stratification by IBD subtype and disease activity, and (iii) evaluation against gold-standard comparators (endoscopy, fecal calprotectin).

### 6.6. Summary Table of Methodologies in Key Studies

A number of representative studies have explored different data types, preprocessing strategies, and AI models for bowel sound analysis. As summarized in Table 8, approaches range from smartphone-based CNN models [43,58], to wearable acoustic spotting combined with gradient boosting [2], and transformer architectures applied to spectrogram inputs [58]. Reported performance metrics vary from ~83% AUC in wearable gradient boosting systems to ~89–90% accuracy or AUC in CNN and transformer models, highlighting both the promise and heterogeneity of current methodologies.

## 7. Discussion

### 7.1. The Unmet Need in IBD Monitoring

Artificial intelligence-based bowel sound analysis has the potential to address the key limitations of current IBD monitoring tools by providing a real-time, non-invasive, and accessible method for detecting disease flares or complications. Unlike endoscopy, fecal calprotectin, or MRI, which are invasive, costly, and subject to delays, AI-enabled high-definition auscultation can continuously monitor bowel sounds using wearable or portable devices, allowing for frequent or even continuous assessment outside of clinical settings [43].

AI algorithms can process complex acoustic features and patterns associated with IBD activity, enabling the detection of changes that may precede clinical symptoms or laboratory abnormalities. This approach could facilitate earlier intervention opportunities by alerting clinicians or patients to subclinical disease activity, potentially reducing the risk of severe flares or complications. Furthermore, integration with digital health platforms supports telemonitoring, empowering remote disease management and reducing the need for in-person visits, which aligns with the demonstrated benefits of digital health and AI in improving quality of life, treatment adherence, and resource utilization in IBD [3,9,55].

While the technology is mature from an engineering perspective, widespread clinical adoption will require standardized methodologies, validation in real-world IBD populations, and integration with existing care pathways [9,55].

Nonetheless, AI-based bowel sound analysis represents a promising adjunct to current monitoring strategies, with the potential to transform IBD management by enabling proactive, patient-centered care [9,55].

While most of the included studies report accuracies in the range of 88–96% and AUC values of ≥0.83, these figures must be interpreted in relation to clinical decision-making thresholds. In practice, diagnostic tools intended for screening or flare exclusion require high sensitivity (≥0.90) to minimize missed cases, a threshold consistently achieved by fecal calprotectin for active disease detection but not yet met by bowel sound AI models. Conversely, tools used to confirm active disease or stratify patients for colonoscopy demand higher specificity (≥0.85) to reduce false positives and unnecessary invasive procedures. Current AI-based bowel sound systems fall short of replacing gold standards such as fecal calprotectin, colonoscopy, or cross-sectional imaging. Still, their reported performance suggests clear potential as a complementary adjunct—particularly for home-based monitoring, early flare detection, or triage in low-resource settings where invasive testing is impractical. Thus, while promising, these models are better positioned as supportive rather than standalone diagnostic tools until prospective validation demonstrates equivalence with established biomarkers.

### 7.2. From Noise to Signal: What AI Has Achieved

AI has enabled a shift in bowel sound analysis from subjective auscultation to objective, quantitative, and automated pattern recognition. Multiple studies demonstrate that deep learning models, particularly convolutional neural networks (CNNs), can achieve high accuracy in bowel sound detection and classification.

For example, a bright T-shirt with embedded microphones, combined with a deep learning-based bowel sound event spotting algorithm and gradient boosting classifier, achieved a mean area under the receiver operating characteristic curve (AUC) of at least 0.83 for distinguishing inflammatory bowel disease (IBD) patients from healthy controls, regardless of whether bowel sounds were manually annotated or detected by AI algorithms. This supports the clinical potential of AI-driven bowel sound analysis for disease classification [5,58].

Smartphone-based CNN models have also demonstrated strong performance, achieving 88.9% accuracy and an F1 score of 0.723 in detecting bowel sounds from recordings in a 100-person cohort, with bowel motility indices showing over 98% correlation with manual annotations. This highlights the feasibility of non-invasive, accessible bowel sound analysis using consumer devices [43].

Other studies confirm that CNN-based and hybrid deep learning approaches can reach or exceed 90% accuracy in bowel sound detection, with high specificity and generalizability across different datasets and clinical scenarios [10,56].

These advances are supported by systematic reviews, which note the excellent future potential of AI-based bowel sound analysis for non-invasive diagnosis and monitoring of gastrointestinal conditions. However, further validation and standardization are needed before widespread clinical adoption [8].

### 7.3. Challenges Limiting Clinical Translation

- Small Sample Sizes and Lack of Generalizability: Most studies to date have included small cohorts (often 16–45 participants), with no published work recruiting more than 100 IBD patients or capturing the full spectrum of disease phenotypes and activity. This severely limits the generalizability and external validity of AI models, as highlighted in recent systematic reviews and original research. Without significant, diverse, and multi-center datasets, models risk overfitting and may not perform reliably in broader clinical populations [5,7].
- Heterogeneous Protocols: Study protocols vary widely—for example, some use fasting recordings, while others capture sounds after meals, and microphone types, sensor placements, and labeling strategies also differ. This lack of methodological standardization hinders reproducibility and cross-study comparison, making it challenging to establish robust, clinically meaningful biomarkers [3]. There is considerable heterogeneity in study design, data acquisition protocols, sensor technologies, feature extraction methods, and AI architectures [3,9,10,15]. This variability complicates direct comparison of results and precludes meta-analysis or pooled diagnostic accuracy estimates. Differences in bowel sound recording duration, sensor placement, and annotation standards further contribute to inconsistent findings and limit reproducibility. The lack of standardized, openly available benchmark datasets with expert-confirmed labels is a major barrier to progress [3,10].
- Model Explainability and Clinician Trust: Most current AI models for bowel sound analysis lack explainability features such as saliency mapping, time-frequency heatmaps, or interpretable outputs. This opacity undermines clinician trust and hinders clinical adoption, as clinicians are less likely to rely on “black box” models for decision-making in the absence of transparent, interpretable outputs. To facilitate the clinical adoption of AI models for bowel sound analysis, it is imperative to integrate explainability techniques that elucidate model decision-making processes. Time-frequency saliency maps can visually highlight specific regions within the acoustic signals that significantly influence model predictions, thereby providing clinicians with insights into the rationale behind classifications [59]. Additionally, Shapley Additive Explanations (SHAP) offer a robust framework for quantifying the contribution of each feature to the model’s output, enhancing transparency and trust [60]. Furthermore, prototype-based explanations can compare current acoustic recordings with representative examples from the training dataset, aiding clinicians in understanding model behavior through familiar instances [61]. Incorporating these explainability methods into clinical workflows—such as through visual dashboards or decision support tools—can bridge the gap between AI model outputs and clinical decision-making, fostering greater acceptance and utilization in patient care.
- Absence of Prospective Clinical Validation: No prospective clinical trials have demonstrated that AI-based bowel sound analysis improves patient outcomes such as reduced flares, hospitalizations, or endoscopies. The lack of outcome-based validation is a significant barrier to clinical integration, as emphasized in reviews of AI in IBD [14,15,55]. Most AI models for bowel sound analysis have been developed and tested on small, single-center cohorts, limiting their generalizability [3,8,10,15,55,62]. There is a notable absence of multi-center, prospective validation studies in diverse patient populations, which is essential for establishing clinical utility and regulatory approval. Most of the existing research has been conducted in controlled settings, and real-world performance—especially in ambulatory or home environments—remains largely untested [3,59,62].
- Privacy and Ethical Considerations: Bowel sound recordings, especially when collected via cloud-based wearable platforms, constitute biometric health data and are subject to privacy regulations such as HIPAA in the US and GDPR in the EU. Key concerns include data security, risk of re-identification, informed consent, and the adequacy of de-identification protocols. Current regulatory frameworks often fail to adequately address the unique risks posed by wearable health data, underscoring the need for enhanced transparency, user control, and robust data protection measures to maintain patient trust and comply with legal requirements [63,64,65].

### 7.4. The Path Forward: Beyond Diagnostics

Future systems that integrate wearable microphones (e.g., Baronetto’s smart shirt), smartphone-based classifiers, real-time AI models (such as BowelRCNN and transformer-based approaches), patient-reported outcomes, and calprotectin levels could enable multi-modal disease monitoring and predictive flare alerts by combining objective, continuous physiologic data with subjective symptoms and established biomarkers. This convergence allows for the detection of subtle changes in bowel sound patterns, correlation with patient-reported symptoms, and biochemical evidence of inflammation, potentially improving the sensitivity and specificity of disease monitoring and flare prediction [5,50,53,61,66].

These platforms could support remote management by providing clinicians with real-time, longitudinal data streams, enabling earlier intervention with more personalized patient care. For example, AI-driven alerts based on abnormal bowel sound patterns, rising calprotectin, and worsening patient-reported outcomes could prompt telemedicine outreach, medication adjustments, or expedited in-person evaluation. The integration of these modalities is anticipated to facilitate closed-loop interventions, such as algorithm-driven recommendations for adjusting biologic therapies or initiating corticosteroids, as discussed in the context of future IBD clinical trial design [50,63,67].

However, there are still significant challenges to overcome—including the need for large, diverse datasets to improve generalizability, standardized protocols for collecting and labeling data, better model explainability to build clinician trust, and rigorous prospective clinical validation to show real impact on patient outcomes [46,50,66].

Privacy and ethical concerns regarding continuous biometric data collection and cloud-based analytics must also be addressed to ensure regulatory compliance [63,67].

### 7.5. Toward an Acoustic Biomarker

**Figure 2:**
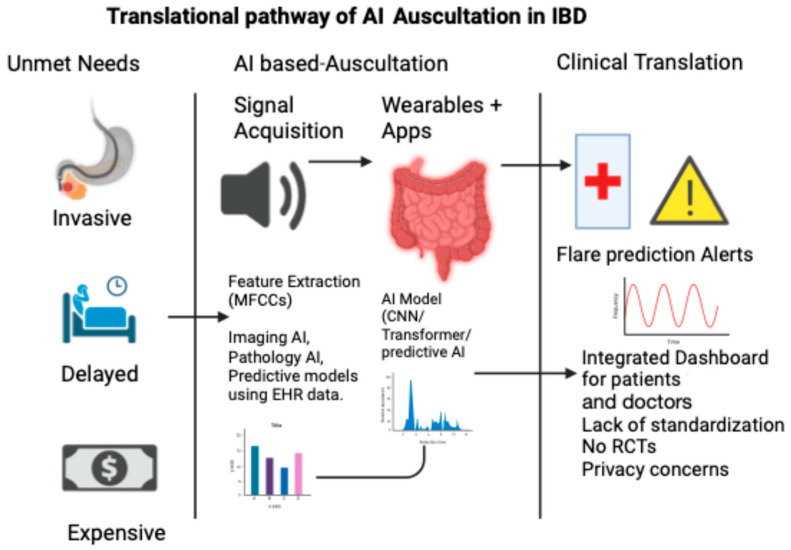
Translational pathway of AI auscultation in IBD.

The concept of an “acoustic biomarker” is no longer speculative. In the same way that echocardiography turns heart sounds into waveforms, AI has the power to convert abdominal murmurs into diagnostic signals. This vision aligns with the goals of precision gastroenterology, in which continuous, real-world data complement episodic imaging or biomarkers. Figure 2 illustrates the translational pathway of AI-based auscultation in IBD, from unmet clinical needs to real-world applications.

“Artificial intelligence does not merely digitize auscultation—it reframes it.”

For bowel sound analysis to achieve clinical relevance, we must align technological capability with clinical need, regulatory rigor, and patient-centered design.

## 8. Results

Artificial intelligence (AI)-powered bowel sound analysis is an emerging, non-invasive modality for diagnosing, characterizing, and managing inflammatory bowel disease (IBD). Recent advances in signal processing and feature extraction—such as Efficient-U-Net for high-resolution event spotting and spectrogram-based convolutional neural networks (CNNs) for classification—enable precise temporal localization and physiologically meaningful feature extraction from continuous, noisy recordings [3,10].

Quantitative Performance Metrics

AI models for bowel sound analysis have demonstrated high diagnostic performance in IBD cohorts. Automated bowel sound analysis using wavelet transformations, multi-layer perceptrons, and autoregressive-moving-average models has achieved diagnostic accuracy rates of 90% or higher in differentiating IBD from healthy controls and other GI disorders [9,10]. Deep learning approaches, including hybrid convolutional and recursive neural networks, have reported accuracy > 93% and specificity > 97% for bowel sound event detection, which is crucial for clinical diagnosis [10]. Smartphone-based CNN models have achieved a bowel sound detection accuracy of 88.9% and F-measure of 72.3% in cross-validation, with bowel motility prediction correlating at over 98% with manual labels [43]. Spectrogram -CBB detector achieved approximately 91% accuracy for bowel sound detection in a 30-participant cohort, with consistent performance across subjects, supporting the feasibility of spectrogram-based deep learning for GI sound analysis [49]. These results are consistent across multiple studies and platforms, supporting the robustness of AI-based acoustic analysis.

Feature Extraction and Clinical Relevance

Key acoustic features—such as Mel-Frequency Cepstral Coefficients (MFCCs), spectral centroid, jitter/shimmer, and entropy—are consistently altered in IBD and irritable bowel syndrome (IBS), and their extraction enables accurate disease characterization [3,9]. The use of large, expertly annotated datasets is essential for model training and benchmarking, supporting generalizability and reproducibility [3].

Clinical Implications

- Diagnosis: High sensitivity and specificity (typically 85–97%) support the use of AI-powered bowel sound analytics as a non-invasive adjunct for IBD diagnosis, potentially reducing reliance on invasive procedures such as colonoscopy and fecal calprotectin testing [3,10,64].
- Disease Activity Monitoring: Real-time, objective assessment of bowel sounds enables continuous disease activity tracking, facilitating early detection of flares and response to therapy [49,62,64,68].
- Treatment Management: Predictive models can stratify patients by risk and forecast response to biologics, supporting personalized treatment decisions [14,55,62,65].
- Resource Optimization: Integration with wearable platforms and remote monitoring technologies can decrease the need for in-person visits, optimize resource utilization, and improve patient quality of life without compromising care standards [55,68].

**Figure 3:**
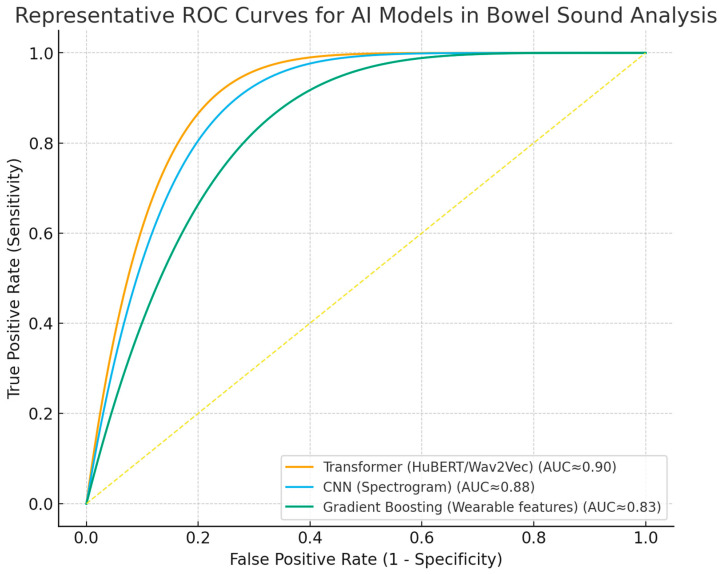
Receiver operating characteristic (ROC) curves for representative AI approaches in bowel sound analysis. Curves illustrate discrimination of transformer, spectrogram-CNN, and gradient boosting (wearable features) models; AUC approximate values reported in the literature. The dashed line indicates no-skill performance.

**Figure 4:**
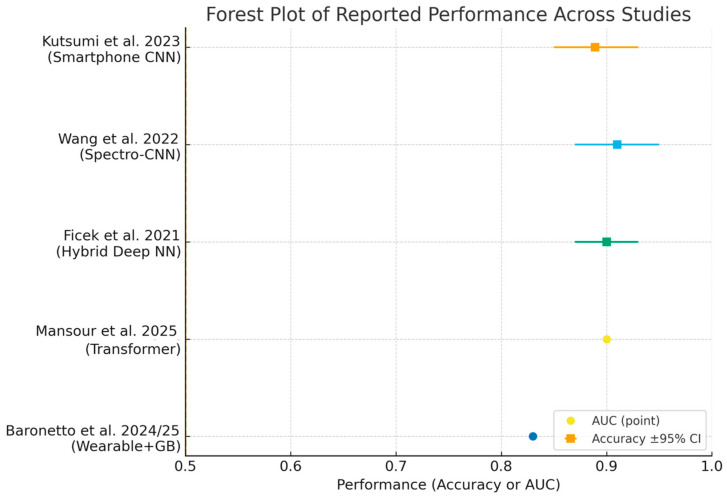
Forest plot summarizing reported performance across included studies [2,10,43,49,57]. Squares show accuracy with 95% Wilson confidence intervals when sample size was available; circles show AUC point estimates when confidence intervals were not reported. Abbreviations: AUC, area under the ROC curve.

Despite these advances, widespread clinical implementation requires further validation in diverse populations, harmonization of analytical methodologies, and integration into digital clinical workflows [14,49,62,64,65]. Ongoing research should prioritize prospective studies, external validation, and the development of standardized reporting frameworks. ROC curves (Figure 3) and forest plots (Figure 4) collectively demonstrate the diagnostic performance and variability of AI models in bowel sound analysis.

While these values demonstrate strong technical feasibility, whether such performance is sufficient for clinical decision-making remains uncertain and is addressed in the Discussion.

## 9. Limitations

Small and homogeneous sample sizes—often comprising 16 to 100 participants, frequently with a predominance of healthy volunteers or pooled patients with Crohn’s disease and ulcerative colitis, without detailed phenotypic stratification—result in models that are highly susceptible to overfitting and lack external validity. This severely limits the generalizability of AI-based bowel sound analysis across diverse populations, including pediatric versus adult patients, those with perianal or ileal-predominant Crohn’s disease, and post-operative states such as ileostomy, as these subgroups are rarely represented or analyzed separately in current studies. As a result, model performance in these underrepresented groups is unknown and likely suboptimal [3,8]. Table 9 summarizes some common limitations.

The absence of gold-standard clinical endpoints—such as endoscopic indices (e.g., Simple Endoscopic Score for Crohn’s Disease, Mayo score), biomarkers (fecal calprotectin, C-reactive protein), and validated clinical activity scores (Harvey–Bradshaw Index)—in most studies prevents robust assessment of whether AI-detected bowel sound patterns truly reflect IBD pathophysiology or merely background motility variations. Without these reference standards, it is not possible to determine the clinical relevance or disease specificity of the detected acoustic features [15].

Inconsistent recording protocols and hardware, including variability in sensor type, placement, recording duration, and patient condition (fasting vs. postprandial), further undermine replicability and comparability across studies, impeding the development of standardized, clinically actionable tools. The lack of external validation and limited model explainability (e.g., absence of interpretable outputs or saliency mapping) hinder clinician trust, slow regulatory approval, and restrict clinical adoption, as highlighted in recent reviews [3,8].

## 10. Conclusions

The current state of artificial intelligence-based bowel sound analysis for inflammatory bowel disease (IBD) demonstrates technical feasibility and early diagnostic potential, particularly with wearable devices and deep learning models. However, translating this into a clinically validated, non-invasive, and explainable monitoring tool still requires several critical steps.

Standardization of protocols is essential, as heterogeneity in sensor types, placement, recording duration, and patient conditions hampers reproducibility and cross-study comparison, making consensus protocols for data acquisition and annotation crucial for robust multi-center studies and regulatory acceptance [3,10,44].

Multi-center data with gold-standard endpoints is needed, as current small, uniform cohorts limit validation of acoustic biomarkers [5,14].

Prospective trials are needed to validate AI-based bowel sound analysis, as no system has yet been tested in real-world workflows or been shown to improve clinical outcomes [5,49,55,66].

Model interpretability is essential for clinician trust and regulatory approval, requiring explainability techniques like saliency mapping or interpretable feature extraction for shared decision-making [5,55].

Compliance with ethical and regulatory standards (HIPAA, GDPR, FDA/CE) is mandatory, especially for wearable and home-based platforms. Privacy, data security, and informed consent are key challenges for continuous biometric monitoring [55,69,70].

The literature highlights significant heterogeneity in sensor types, placement, recording duration, and patient conditions, which impedes reproducibility and cross-study comparison. Establishing consensus protocols for data acquisition and annotation is a prerequisite for robust multi-center studies and regulatory acceptance [3,12,67].

Multi-center data collection, with linkage to endoscopic, imaging, and biomarker data, is necessary to ensure generalizability and clinical relevance. Most studies to date have small, homogeneous cohorts and lack gold-standard endpoints, such as endoscopic scores or fecal calprotectin, which limits the ability to validate acoustic biomarkers against actual disease activity [50,71].

Prospective clinical trial validation is necessary to demonstrate that AI-based bowel sound analysis can accurately predict flares, monitor therapy response, and ultimately improve patient outcomes. No current system has been tested in real-world workflows or been shown to impact clinical endpoints [15,50,71,72].

Model interpretability must be addressed to build clinician trust and facilitate regulatory approval. Incorporating explainability techniques—such as saliency mapping or interpretable feature extraction—will be crucial for integration into shared decision-making [49,65,69,70].

## Figures and Tables

**Table 1:** Clinical Bowel Sounds Pattern in IBD.

Sound Type	Acoustic Description	Pathophysiological Basis	Associated IBD Feature
Normal	Soft gurgles every 5–15 s	Balanced peristalsis and segmentation	Remission
Hyperactive	Loud, frequent, high-pitched	Inflammation, diarrhea, stricture stress	Flare-up, diarrhea, early obstruction
Hypoactive	Infrequent, low intensity	Myenteric suppression, ileus	Post-op, toxic megacolon, fibrosis
Absent	Silent abdomen >5 min	Paralytic ileus, perforation, ischemia	Advanced complication
Tinkling	Sharp, metallic, intermittent	Partial obstruction, narrow lumen	Crohn’s strictures, adhesions
Cramping/Gurgling	Irregular bursts of pain	Spasmodic contractions, inflammation	Active UC or CD

**Table 2:** Some common characteristics and BS pattern.

BS Pattern	Characteristics	IBD Context
Normal	Soft gurgles every 5–15 s	Usually seen during remission
Hyperactive/High-pitched	Frequent, loud gurgles	Associated with flare-ups, strictures, diarrhea
Hypoactive/Absent	Softened or silenced	Indicates ileus, post-op state, obstruction, perforation
Cramping/Gurgling	Irregular with discomfort	Seen in active inflammation and spasms

**Table 3:** Summary of the study model with its results.

Study/Model	Dataset and Platform	Task	Results
Kutsumi et al. (2023, Smartphone CNN) [43]	77–100 users; phone mic	BS detection	Acc = 88.9%, F_1_ = 0.723; SSI correlate > 98%
BowelRCNN (2025, RCNN) [56]	19 patients (60 min audio)	BS detection	Acc = 96%, F_1_ = 0.71
Mansour et al. (2025, Transformer) [57]	16 healthy, spectrograms	BS detection/classification	AUC = 0.89
Baronetto et al. (2023, EffUNet + GB) [47]	24 IBD/21 control, smart T-shirt	IBD vs. control classification	AUC ≥ 0.83, robust to noise

**Table 4:** Summary of the study and its sample size.

Study	Sample Size	Population
Craine et al., 1999 [1]	24	IBS, Crohn’s, controls
Baronetto et al., 2024 [2]	45	IBD (CD, UC), controls
Kutsumi et al., 2023 [43]	100	Mixed general population

**Table 5:** Summary of the data acquisition methods.

Method	Devices Used	Protocol Length	Sites Recorded
Smartphone microphone	Katsumi et al. [43]	5–30 min	Lower abdomen
Bright T-shirt (8-mic array)	Baronetto et al. [2]	60–120 min	Entire abdomen

**Table 6:** Summary of the Preprocessing and Feature Extraction.

Study	Event Spotting	Features Used
Baronetto et al. [2]	EffUNet	Frequency, entropy
Mansour et al. [57]	Manual/Auto	MFCCs, S–S intervals

**Table 7:** Summary of the Training Approaches.

Model Type	Studies Using It	Reported Metrics
Logistic regression, SVM	Craine et al. [1]	Acc = 85–90%
CNN (spectrogram input)	Kutsumi et al. [43]	Acc = 88.9–91%
Region-based CNN (BowelRCNN)	Nowak et al. [3]	F1 = 0.71
Transformer (HuBERT, Wav2Vec)	Mansour et al. [57]	AUC = 0.89
Gradient boosting of T-shirt data	Baronetto et al. [2]	AUC ≥ 0.83

**Table 8:** Summary Table of Methodologies in Key Studies.

Study	Data Type	Preprocessing	Model	Target Task	Metric
Craine et al. [1]	S–S interval	Manual annotation	Logistic regression	IBS vs. Crohn	Acc = 85–90%
Katsumi et al. [43]	Raw audio (smartphone)	STFT, normalization	CNN	BS detection	Acc = 88.9%
Baronetto et al. [2]	Multi-mic audio (wearable)	EffUNet spotting	GBM	IBD vs. Control	AUC ≥ 0.83
Mansour et al. [57]	Spectrogram	Auto spotting	Transformer	BS classification	AUC = 0.89

**Table 9:** Summary of common limitations.

Limitation	Description
Small sample size	Most studies < 100 subjects
Lack of IBD-specific outcomes	Rare correlation with endoscopy, calprotectin
Variable protocols	Device, site, time-inconsistent
No external validation	No multi-center or prospective trials
No explainability	CNNs/transformers used as black boxes
Regulatory uncertainty	No FDA or CE approvals yet

## Data Availability

No new data were created or analyzed in this study.
